# Redetermination of di­ammonium trivanadate, (NH_4_)_2_V_3_O_8_

**DOI:** 10.1107/S2414314620004885

**Published:** 2020-04-09

**Authors:** Aarón Pérez-Benítez, Sylvain Bernès

**Affiliations:** a Facultad de Ciencias Químicas, Benemérita Universidad Autónoma de Puebla, Av. San Claudio y 18 Sur, 72570 Puebla, Pue., Mexico; b Instituto de Física, Benemérita Universidad Autónoma de Puebla, Av. San Claudio y 18 Sur, 72570 Puebla, Pue., Mexico; Vienna University of Technology, Austria

**Keywords:** crystal structure, vanadate, ammonium, high-resolution data, redetermination

## Abstract

The crystal structure of (NH_4_)_2_V_3_O_8_ has been reported twice using single-crystal X-ray data [Theobald *et al.* (1984 ▸). *J. Phys. Chem. Solids*, **45**, 581–587; Range *et al.* (1988 ▸). *Z. Naturforsch. Teil B*, **43**, 309–317]. In both cases, the orientation of the ammonium cation in the asymmetric unit was poorly defined: in Theobald’s study, the shape and dimensions were constrained for NH_4_^+^, while in Range’s study, H atoms were not included. In the present study, we collected a highly redundant data set for this ternary oxide, at 0.61 Å resolution, using Ag *K*α radiation. These accurate data reveal that the NH_4_^+^ cation is disordered by rotation around a non-crystallographic axis. The rotation axis coincides with one N—H bond lying in the mirror *m* symmetry element of space-group type *P*4*bm*, and the remaining H sites were modelled over two disordered positions, with equal occupancy. It therefore follows that the NH_4_^+^ cations filling the space available in the (001) layered structure formed by (V_3_O_8_)^2–^ ions do not form strong N—H⋯O hydrogen bonds with the mixed-valent oxidovanadate(IV,V) anions. This feature could have consequences for the Li-ion inter­calation properties of this material, which is used as a cathode for supercapacitors.

## Abstract 2

The crystal structure of (NH_4_)_2_V_3_O_8_ has been redetermined using data collected at 0.61 Å resolution, showing that the ammonium cation is disordered by rotation around a non-crystallographic axis.

## Graphical abstract

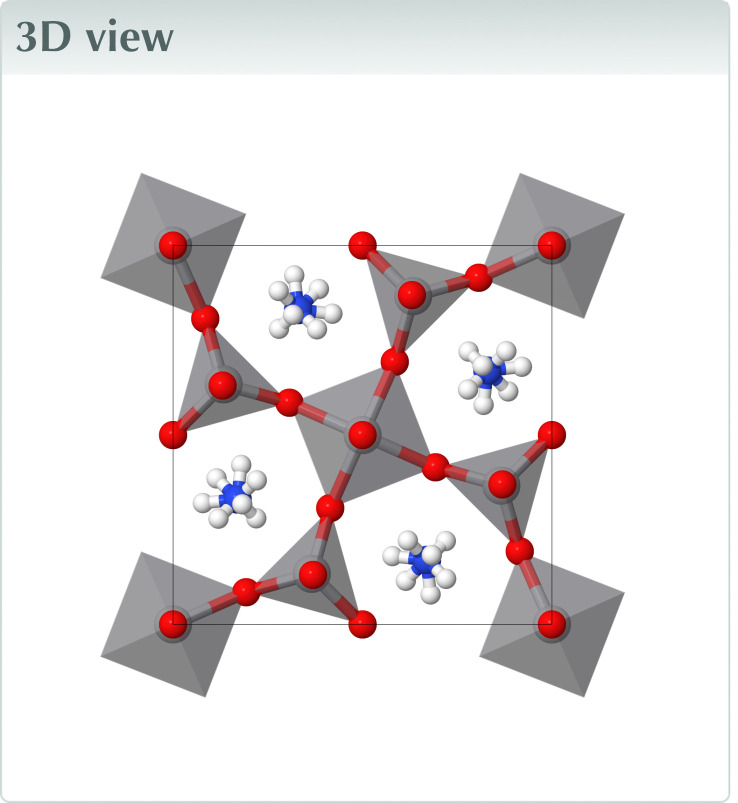

## Structure description

Di­ammonium trivanadate, (NH_4_)_2_V_3_O_8_, is a well-studied mixed-valent ternary vanadium(IV,V) oxide, in particular for the building of cathodes for supercapacitors, including lithium-ion batteries. Of particular inter­est is its very high specific capacity, which could theoretically reach 442 mA h/g, with a Coulombic efficiency close to 100% (Xu *et al.*, 2016 ▸). Moreover, it can be obtained cheaply and simply, for example by hydro­thermal reduction of NH_4_VO_3_ (Ren *et al.*, 2007 ▸), by electroreduction of NH_4_VO_3_ (Andrukaitis *et al.*, 1990 ▸), or by solid-state reaction between NH_4_VO_3_ and V_2_O_3_ at low pressure (Liu & Greedan, 1995 ▸).

This mixed-valence oxide belongs to an isotypic series of *A*_2_V_3_O_8_ compounds (*A* = K, Rb, Cs, NH_4_; Yeon *et al.*, 2013 ▸) adopting the crystal structure of fresnoite, a pyrosilicate mineral with formula Ba_2_TiSi_2_O_8_. Anions (V_3_O_8_)^2–^ form a layered structure extending parallel to (001), based on [V^V^O_4_] and [V^IV^O_5_] polyhedra sharing oxygen atoms, while NH_4_^+^ cations are sandwiched by the anionic layers (The Materials Project, 2019 ▸). The crystal structure in space group *P*4*bm* has been determined at least twice by single-crystal X-ray diffraction. The first report (Theobald *et al.*, 1984 ▸) is based on X-ray data collected on a PW-1100 diffractometer, up to 0.62 Å resolution, with a rather large crystal, with dimensions 0.45×0.30×0.03 mm^3^. The refinement seems to be of very good quality. However, the authors mention that H-atom positions for the cation NH_4_^+^ retrieved from a difference map did not result in a satisfactory refinement, so the shape and dimensions were constrained for the cation. The second independent report (Range *et al.*, 1988 ▸) is based on X-ray data at even higher resolution, measured on a CAD-4 diffractometer. However, H-atom positions were not included for this refinement. For both refinements, only one octant of the reciprocal space was collected [0 ≤ *h* ≤ *h*_max_, 0 ≤ *k* ≤ *k*_max_ and 0 ≤ *l* ≤ *l*_max_], a common practice in the 1980s. This, however, precludes an accurate correction of data for absorption and other crystal-shape-related effects. A third article published in 2007 mentioned a single-crystal X-ray study for (NH_4_)_2_V_3_O_8_, using a very small plate-shaped crystal with dimensions 0.04×0.03×0.004 mm^3^, collected on an IPDS diffractometer equipped with a rotating anode (Ren *et al.*, 2007 ▸). Apparently, H atoms were included, but details about the structure were not provided in this article.

**Figure 1:**
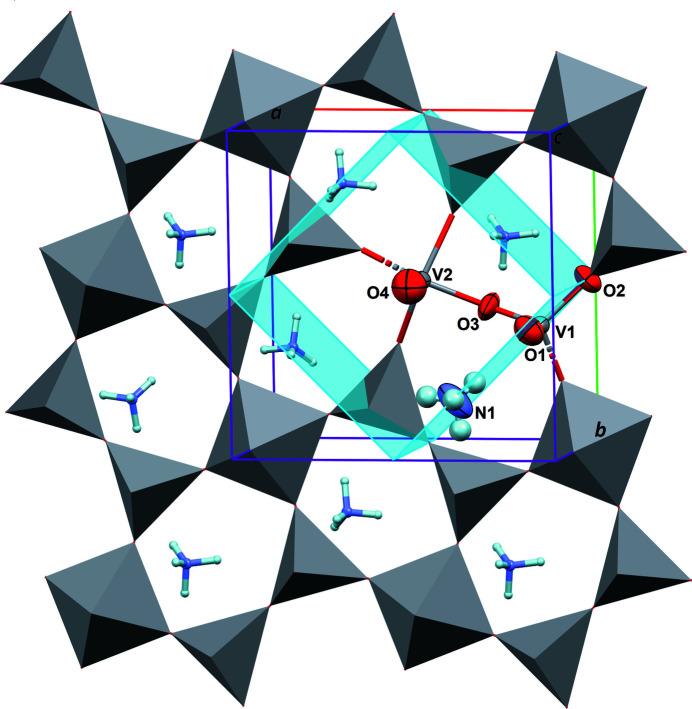
Crystal structure of (NH_4_)_2_V_3_O_8_ viewed approximately down the *c* axis. The asymmetric unit is represented with displacement ellipsoids drawn at the 90% probability level, and other vanadate groups are drawn with a polyhedral representation. Only one (V_3_O_8_)^2–^ layer normal to [001] is represented, and a single position for the disordered NH_4_^+^ cation has been accentuated. Blue planes are the mirror *m* elements of space-group type *P*4*bm*.

We have now redetermined the crystal structure of (NH_4_)_2_V_3_O_8_ (Fig. 1 ▸), after collecting a highly redundant data set at 295 K: redundancy was 33 for a resolution of 0.61 Å. The dimensions of the vanadium oxide layers are remarkably close to those determined by Theobald *et al.* (1984 ▸), apart for the axial bond lengths V1=O1 and V2=O4, which were overestimated by *ca* 0.02–0.06 Å (see comparison in Table 1 ▸). This difference could be a consequence of the wrong positions of some H atoms in Theobald’s model.

**Figure 2:**
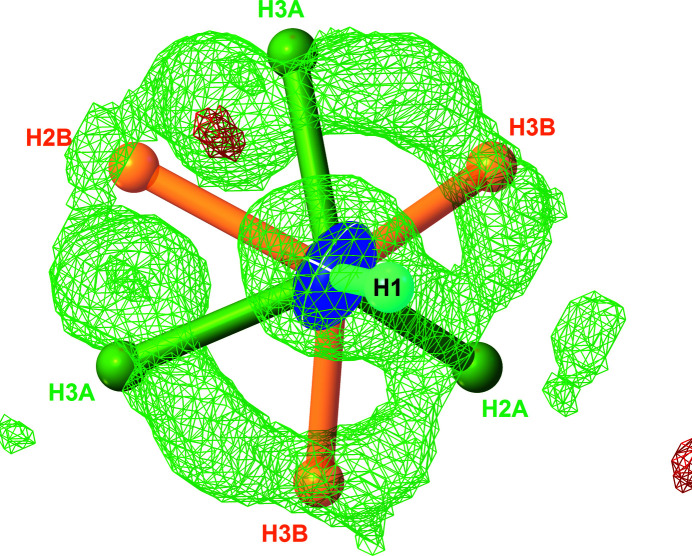
Difference electron-density map in the vicinity of the N-atom site calculated on the basis of a model including N1 and H1 atoms (*R*_1_ = 0.0183, *wR*_2_ = 0.0420). The difference map is plotted at the 0.18 e^−^ Å^−3^ level with a resolution of 0.05 Å (Dolomanov *et al.*, 2009 ▸). At this level, only positive residuals are observed (green wire), corresponding essentially to the N—H *σ* bond and missing H atoms. Positions for all H atoms in the final refinement (*R*_1_ = 0.0163, *wR*_2_ = 0.0296) are superimposed on the calculated difference map, showing the fit between the experimental data and the proposed model.

We identified that the NH_4_^+^ cation is disordered by rotation around a non-crystallographic axis. The N atom lies in the mirror plane *m* of space group *P*4*bm* (Wyckoff position 4*c*), and after refining positions and displacement parameters for all non-H atoms, the highest positive residual electron density is found in the same plane and can be refined as an H atom (H1). The subsequent difference map suggests that the three missing H atoms are continuously disordered along a ring normal to the *m* plane (Fig. 2 ▸). The best model was eventually reached with the second ammonium H atom equally disordered over two 4*c* positions (H2*A* and H2*B*), and the last H atom placed in a general position (8*d*), also disordered over two sites, H3*A* and H3*B*, with occupancies of 0.5 (Fig. 2 ▸). Both NH_4_^+^ parts were refined with soft restraints (see *Refinement details*). The correctness of the model is validated through the refinement of isotropic displacement parameters for all H atoms. Any ordered model for H2 and H3 converged towards too high *U*_iso_ parameters, in the range 0.12 to 0.18 Å^2^, while *U*_iso_(H1) ≃ 0.05 Å^2^. Moreover, N—H bond lengths refined around 0.74 Å, whereas a bond length close to 0.80 Å is expected. In contrast, the proposed model has refined *U*_iso_(H) parameters in the range 0.051 (12) to 0.10 (4) Å^2^ and N—H bond lengths between 0.78 (3) and 0.83 (3) Å.

**Figure 3:**
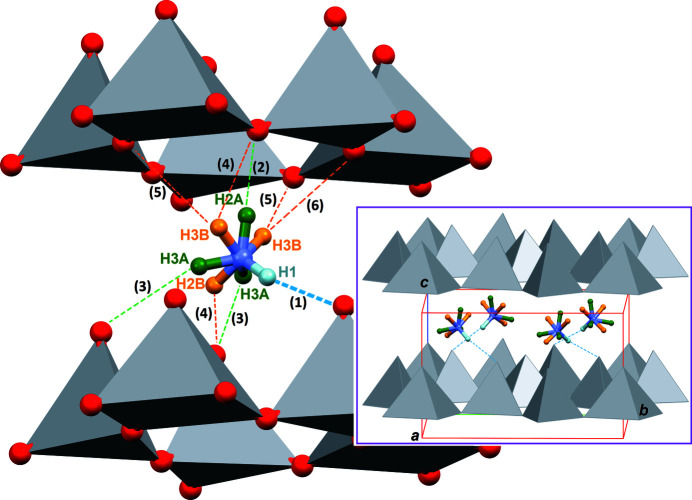
Part of the crystal structure of (NH_4_)_2_V_3_O_8_ showing the N—H⋯O hydrogen bonds. The NH_4_^+^ cation is disordered over two positions, N1/H1/H2*A*/H3*A* (green) and N1/H1/H2*B*/H3*B* (orange). Hydrogen bonds are represented as dashed lines, with a label referring to entries in Table 2 ▸. The inset shows how the cations inter­act with the vanadium oxide matrix. Only the strongest N1—H1⋯O1 hydrogen bond is represented.

As a consequence, only one significant N—H⋯O hydrogen bond of medium strength is formed in the crystal structure, involving the N1—H1 bond, which is also the non-crystallographic rotation axis for the disordered cation (Table 2 ▸, first entry). All other N—H⋯O contacts are weaker, with H⋯O separations in the range 2.24 (3)–2.49 (3) Å (Table 2 ▸, entries 2–6; Fig. 3 ▸). This makes a difference, for instance, with the structure of ammonium metavanadate, NH_4_VO_3_, for which the ammonium cation is ordered and which forms at least two strong hydrogen bonds with the vanadium oxide matrix (Pérez-Benítez & Bernès, 2018 ▸). The rather poor inter­action of the ammonium cation with the (V_3_O_8_)^2−^ layers in the crystal structure of (NH_4_)_2_V_3_O_8_ could be of inter­est for its application as a cathode material for supercapacitors, since the replacement of NH_4_^+^ cations by Li^+^ should be a process with a low free enthalpy, compared to that of other fresnoite-type vanadates. From the structural point of view, however, the matter is more complex: although no definitive data are available so far, it seems that Li_2_V_3_O_8_ does not belong to the fresnoite structural type. Theoretical (Koval’chuk *et al.*, 2002 ▸) and experimental (de Picciotto *et al.*, 1993 ▸; Jouanneau *et al.*, 2005 ▸) data for Li_1+*x*_V_3_O_8_ show that these vanadates crystallize in the hewettite structural type, in space-group type *P*2_1_/*m*, as does Na_2_V_3_O_8_ (Bachmann & Barnes, 1962 ▸). On the other hand, to the best of our knowledge, no studies have been made hitherto on the pseudo-binary system Li_2_V_3_O_8_–(NH_4_)_2_V_3_O_8_.

## Synthesis and crystallization

Good-quality single crystals of (NH_4_)_2_V_3_O_8_ were obtained as a by product during the reaction between ammonium metavanadate (NH_4_VO_3_, 0.5 g, 4.27 mmol), and metformin hydro­chloride (HMetf^+^Cl^−^, 0.425 g, 2.56 mmol) in 75 ml of distilled water and 1 ml of acetic acid 5% *v*/*v*. Ammonium metavanadate and metformin hydro­chloride were dissolved in water by gently heating the mixture. Given that our main purpose was to synthesize deca­vanadate salts (HMetf)_6_(V_10_O_28_)·*n*(H_2_O), a small amount of acetic acid was added to the mixture, to achieve a pH of *ca* 6.5. In fact, under these conditions, a mixture of two different hydrates of the desired compound were obtained, (HMetf)_6_(V_10_O_28_)·6(H_2_O) (orange needles) and (HMetf)_6_(V_10_O_28_)·4(H_2_O) (orange plates). Other crystallized products were the double salt (NH_4_)_2_(H_2_Metf)_2_(V_10_O_28_)·10(H_2_O) (orange needles), the unreacted colourless HMetf^+^Cl^−^, and a tiny amount of dark-blue single-crystals of (NH_4_)_2_V_3_O_8_, the structure of which is discussed here.

## Refinement

Crystal data, data collection and structure refinement details are summarized in Table 3 ▸. The five H atoms modelling the disordered NH_4_^+^ cation were refined with free coordinates and free isotropic displacement parameters. While H1 fully occupies its site, H2 and H3 are equally disordered over two sites, H2*A*/H2*B* and H3*A*/H3*B*, respectively. All N—H bond lengths were restrained to a common free variable *d*, with a standard deviation of 0.02 Å (5 restraints), and the tetra­hedral shape of each disordered part was upheld by restricting H⋯H separations to (8/3)^1/2^×*d*, within a standard deviation of 0.03 Å (12 restraints). The free variable *d* converged to 0.81 (2) Å (Sheldrick, 2015*b* ▸). The crystal was considered as a racemic twin, and the batch scale factor refined to *x* = 0.36 (4).

## Supplementary Material

Crystal structure: contains datablock(s) I. DOI: 10.1107/S2414314620004885/wm4127sup1.cif

Structure factors: contains datablock(s) I. DOI: 10.1107/S2414314620004885/wm4127Isup2.hkl

CCDC references: 1997397, 1997400, 1997401

Additional supporting information:  crystallographic information; 3D view; checkCIF report

## Figures and Tables

**Table 1:** Bond lengths (Å) and angles (°) in (NH_4_)_2_V_3_O_8_ for vanadium sites determined in this work, compared to those reported in previous studies Labelling scheme for atomic sites is that used in the present work.

Parameter	1984 study *^*a*^*	1988 study *^*b*^*	This work
Bond lengths (Å)			
V1—O1	1.660 (5)	1.618	1.6353 (18)
V1—O2	1.793 (2)	1.803	1.7958 (9)
V1—O3 (×2)	1.709 (3)	1.720	1.7123 (12)
V2—O3 (×4)	1.962 (3)	1.972	1.9647 (12)
V2—O4	1.650 (8)	1.576	1.592 (3)
			
Bond angles (°)			
O1—V1—O2	109.2 (3)	110.1	109.20 (10)
O1—V1—O3 (×2)	111.3 (3)	112.2	111.16 (6)
O3—V1—O2 (×2)	107.9 (3)	106.9	107.89 (7)
O3—V1—O3	109.2 (3)	108.1	109.42 (8)
O3—V2—O3 (×2)	146.1 (2)	143.9	145.99 (10)
O3—V2—O3 (×4)	85.1 (1)	84.5	85.09 (3)
O4—V2—O3 (×4)	107.0 (1)	108.0	107.01 (5)

Notes: (*a*) Theobald *et al.* (1984 ▸); (*b*) Range *et al.* (1988 ▸); parameters for this refinement were calculated from the published unit-cell parameters and atom coordinates.

**Table 2:** Hydrogen-bond geometry (Å, °)

*D*—H⋯*A*	*D*—H	H⋯*A*	*D*⋯*A*	*D*—H⋯*A*
N1—H1⋯O1	0.78 (3)	2.07 (3)	2.842 (3)	168 (3)
N1—H2*A*⋯O3^i^	0.81 (3)	2.41 (4)	2.980 (3)	129 (4)
N1—H3*A*⋯O1^ii^	0.83 (3)	2.24 (3)	3.006 (2)	153 (4)
N1—H2*B*⋯O1^iii^	0.81 (3)	2.37 (2)	3.006 (3)	136 (1)
N1—H3*B*⋯O3^iv^	0.81 (3)	2.49 (3)	3.274 (3)	162 (4)
N1—H3*B*⋯O3^v^	0.81 (3)	2.39 (4)	2.980 (3)	130 (4)

Symmetry codes: (i) 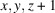 ; (ii) 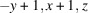 ; (iii) 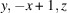 ; (iv) 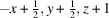 ; (v) 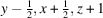 .

**Table 3:** Experimental details

Crystal data	
Chemical formula	(NH_4_)_2_V_3_O_8_
*M*_r_	316.90
Crystal system, space group	Tetragonal, *P*4*b* *m*
Temperature (K)	295
*a*, *c* (Å)	8.9062 (4), 5.5784 (3)
*V* (Å^3^)	442.48 (5)
*Z*	2
Radiation type	Ag *K*α, λ = 0.56083 Å
μ (mm^−1^)	1.60
Crystal size (mm)	0.06 × 0.06 × 0.03

Data collection	
Diffractometer	Stoe Stadivari
Absorption correction	Multi-scan (*X-RED32*; Stoe & Cie, 2019 ▸)
*T*_min_, *T*_max_	0.410, 1.000
No. of measured, independent and observed [*I* > 2σ(*I*)] reflections	26907, 1106, 930
*R*_int_	0.061
(sin θ/λ)_max_ (Å^−1^)	0.823

Refinement	
*R*[*F* ^2^ > 2σ(*F* ^2^)], *wR*(*F* ^2^), *S*	0.016, 0.030, 0.87
No. of reflections	1106
No. of parameters	57
No. of restraints	18
H-atom treatment	All H-atom parameters refined
Δρ_max_, Δρ_min_ (e Å^−3^)	0.44, −0.23
Absolute structure	Refined as an inversion twin.
Absolute structure parameter	0.36 (4)

Computer programs: *X-AREA* (Stoe & Cie, 2019 ▸), *SHELXT2018* (Sheldrick, 2015*a* ▸), *SHELXL2018* (Sheldrick, 2015*b* ▸), *Mercury* (Macrae *et al.*, 2020 ▸) and *publCIF* (Westrip, 2010 ▸).

## full crystallographic data

### Crystal data

**Table:**

(NH_4_)_2_V_3_O_8_	*D*_x_ = 2.379 Mg m^−^^3^
*M**_r_* = 316.90	Ag *K*α radiation, λ = 0.56083 Å
Tetragonal, *P*4*b**m*	Cell parameters from 21170 reflections
*a* = 8.9062 (4) Å	θ = 2.9–32.5°
*c* = 5.5784 (3) Å	µ = 1.60 mm^−^^1^
*V* = 442.48 (5) Å^3^	*T* = 295 K
*Z* = 2	Prism, blue
*F*(000) = 310	0.06 × 0.06 × 0.03 mm

### Data collection

**Table:**

Stoe Stadivari diffractometer	1106 independent reflections
Radiation source: Sealed X-ray tube, Axo Astix-f Microfocus source	930 reflections with *I* > 2σ(*I*)
Graded multilayer mirror monochromator	*R*_int_ = 0.061
Detector resolution: 5.81 pixels mm^-1^	θ_max_ = 27.5°, θ_min_ = 2.6°
ω scans	*h* = −14→14
Absorption correction: multi-scan (X-RED32; Stoe & Cie, 2019)	*k* = −14→14
*T*_min_ = 0.410, *T*_max_ = 1.000	*l* = −9→9
26907 measured reflections	

### Refinement

**Table:**

Refinement on *F*^2^	Secondary atom site location: difference Fourier map
Least-squares matrix: full	Hydrogen site location: difference Fourier map
*R*[*F*^2^ > 2σ(*F*^2^)] = 0.016	All H-atom parameters refined
*wR*(*F*^2^) = 0.030	*w* = 1/[σ^2^(*F*_o_^2^) + (0.0141*P*)^2^] where *P* = (*F*_o_^2^ + 2*F*_c_^2^)/3
*S* = 0.87	(Δ/σ)_max_ < 0.001
1106 reflections	Δρ_max_ = 0.44 e Å^−^^3^
57 parameters	Δρ_min_ = −0.23 e Å^−^^3^
18 restraints	Absolute structure: Refined as an inversion twin.
0 constraints	Absolute structure parameter: 0.36 (4)
Primary atom site location: dual	

### Special details

**Table:**

Refinement. Refined as a 2-component inversion twin.

### Fractional atomic coordinates and isotropic or equivalent isotropic displacement parameters (Å^2^)

**Table:**

	*x*	*y*	*z*	*U*_iso_*/*U*_eq_	Occ. (<1)
V1	0.13352 (2)	0.63352 (2)	0.29279 (12)	0.01254 (6)	
V2	0.500000	0.500000	0.28825 (18)	0.01385 (9)	
O1	0.13050 (15)	0.63050 (15)	0.5859 (3)	0.0251 (4)	
O2	0.000000	0.500000	0.1799 (4)	0.0208 (5)	
O3	0.30693 (14)	0.58500 (14)	0.1852 (3)	0.0237 (2)	
O4	0.500000	0.500000	0.5737 (5)	0.0326 (6)	
N1	0.33009 (19)	0.83009 (19)	0.8234 (6)	0.0309 (6)	
H1	0.280 (2)	0.780 (2)	0.740 (5)	0.051 (12)*	
H2A	0.317 (5)	0.817 (5)	0.965 (6)	0.10 (4)*	0.5
H3A	0.320 (3)	0.921 (3)	0.794 (9)	0.08 (2)*	0.5
H2B	0.385 (3)	0.885 (3)	0.749 (11)	0.06 (3)*	0.5
H3B	0.279 (4)	0.880 (4)	0.914 (6)	0.09 (2)*	0.5

### Atomic displacement parameters (Å^2^)

**Table:**

	*U*^11^	*U*^22^	*U*^33^	*U*^12^	*U*^13^	*U*^23^
V1	0.00932 (8)	0.00932 (8)	0.01900 (14)	−0.00001 (10)	0.00065 (19)	0.00065 (19)
V2	0.00970 (11)	0.00970 (11)	0.0222 (2)	0.000	0.000	0.000
O1	0.0275 (6)	0.0275 (6)	0.0202 (8)	−0.0048 (7)	−0.0022 (5)	−0.0022 (5)
O2	0.0208 (7)	0.0208 (7)	0.0208 (12)	−0.0085 (9)	0.000	0.000
O3	0.0126 (5)	0.0218 (6)	0.0365 (6)	0.0056 (5)	0.0016 (5)	0.0049 (5)
O4	0.0356 (10)	0.0356 (10)	0.0266 (15)	0.000	0.000	0.000
N1	0.0352 (8)	0.0352 (8)	0.0225 (17)	−0.0173 (9)	0.0000 (7)	0.0000 (7)

### Geometric parameters (Å, º)

**Table:**

V1—O1	1.6353 (18)	V2—O4	1.592 (3)
V1—O2	1.7958 (9)	N1—H1	0.78 (3)
V1—O3	1.7123 (12)	N1—H2A	0.81 (3)
V1—O3^i^	1.7123 (12)	N1—H3A	0.83 (3)
V2—O3	1.9647 (12)	N1—H3A^i^	0.83 (3)
V2—O3^ii^	1.9647 (12)	N1—H2B	0.81 (3)
V2—O3^iii^	1.9647 (12)	N1—H3B	0.81 (3)
V2—O3^iv^	1.9647 (12)	N1—H3B^i^	0.81 (3)
			
O1—V1—O2	109.20 (10)	O4—V2—O3	107.01 (5)
O1—V1—O3	111.16 (6)	V1—O2—V1^v^	138.94 (15)
O1—V1—O3^i^	111.16 (6)	V1—O3—V2	141.64 (9)
O3—V1—O2	107.89 (7)	H1—N1—H2A	115 (5)
O3^i^—V1—O2	107.89 (7)	H1—N1—H3A	112 (3)
O3—V1—O3^i^	109.42 (8)	H2A—N1—H3A	109 (3)
O3^ii^—V2—O3^iii^	145.99 (10)	H1—N1—H3A^i^	112 (3)
O3^ii^—V2—O3^iv^	85.09 (3)	H2A—N1—H3A^i^	109 (3)
O3^iii^—V2—O3^iv^	85.09 (3)	H3A—N1—H3A^i^	99 (4)
O3^ii^—V2—O3	85.09 (3)	H1—N1—H2B	113 (5)
O3^iii^—V2—O3	85.09 (3)	H1—N1—H3B	111 (3)
O3^iv^—V2—O3	145.99 (10)	H2B—N1—H3B	109 (3)
O4—V2—O3^ii^	107.01 (5)	H1—N1—H3B^i^	111 (3)
O4—V2—O3^iii^	107.01 (5)	H2B—N1—H3B^i^	109 (3)
O4—V2—O3^iv^	107.01 (5)	H3B—N1—H3B^i^	103 (5)
			
O1—V1—O2—V1^v^	0.000 (1)	O1—V1—O3—V2	11.34 (15)
O3—V1—O2—V1^v^	120.94 (6)	O3^i^—V1—O3—V2	134.50 (8)
O3^i^—V1—O2—V1^v^	−120.94 (6)	O2—V1—O3—V2	−108.36 (14)

Symmetry codes: (i) *y*−1/2, *x*+1/2, *z*; (ii) −*y*+1, *x*, *z*; (iii) *y*, −*x*+1, *z*; (iv) −*x*+1, −*y*+1, *z*; (v) −*x*, −*y*+1, *z*.

### Hydrogen-bond geometry (Å, º)

**Table:**

*D*—H···*A*	*D*—H	H···*A*	*D*···*A*	*D*—H···*A*
N1—H1···O1	0.78 (3)	2.07 (3)	2.842 (3)	168 (3)
N1—H2*A*···O3^vi^	0.81 (3)	2.41 (4)	2.980 (3)	129 (4)
N1—H3*A*···O1^vii^	0.83 (3)	2.24 (3)	3.006 (2)	153 (4)
N1—H2*B*···O1^iii^	0.81 (3)	2.37 (2)	3.006 (3)	136 (1)
N1—H3*B*···O3^viii^	0.81 (3)	2.49 (3)	3.274 (3)	162 (4)
N1—H3*B*···O3^ix^	0.81 (3)	2.39 (4)	2.980 (3)	130 (4)

Symmetry codes: (iii) *y*, −*x*+1, *z*; (vi) *x*, *y*, *z*+1; (vii) −*y*+1, *x*+1, *z*; (viii) −*x*+1/2, *y*+1/2, *z*+1; (ix) *y*−1/2, *x*+1/2, *z*+1.
